# Rapid construction of metabolic models for a family of Cyanobacteria using a multiple source annotation workflow

**DOI:** 10.1186/1752-0509-7-142

**Published:** 2013-12-27

**Authors:** Thomas J Mueller, Bertram M Berla, Himadri B Pakrasi, Costas D Maranas

**Affiliations:** 1 Department of Chemical Engineering, The Pennsylvania State University, University Park, Pennsylvania, USA; 2 Department of Energy, Environmental, and Chemical Engineering, Washington University, St. Louis, Missouri, USA; 3 Department of Biology, Washington University, St. Louis, Missouri, USA

**Keywords:** Semi-automated metabolic network reconstruction, Metabolic modeling, Genome-scale metabolic model, Cyanobacteria, Cyanothece

## Abstract

**Background:**

Cyanobacteria are photoautotrophic prokaryotes that exhibit robust growth under diverse environmental conditions with minimal nutritional requirements. They can use solar energy to convert CO_2_ and other reduced carbon sources into biofuels and chemical products. The genus *Cyanothece* includes unicellular nitrogen-fixing cyanobacteria that have been shown to offer high levels of hydrogen production and nitrogen fixation. The reconstruction of quality genome-scale metabolic models for organisms with limited annotation resources remains a challenging task.

**Results:**

Here we reconstruct and subsequently analyze and compare the metabolism of five *Cyanothece* strains, namely *Cyanothece* sp. PCC 7424, 7425, 7822, 8801 and 8802, as the genome-scale metabolic reconstructions *i*Cyc792, *i*Cyn731, *i*Cyj826, *i*Cyp752, and *i*Cyh755 respectively. We compare these phylogenetically related *Cyanothece* strains to assess their bio-production potential. A systematic workflow is introduced for integrating and prioritizing annotation information from the Universal Protein Resource (Uniprot), NCBI Protein Clusters, and the Rapid Annotations using Subsystems Technology (RAST) method. The genome-scale metabolic models include fully traced photosynthesis reactions and respiratory chains, as well as balanced reactions and GPR associations. Metabolic differences between the organisms are highlighted such as the non-fermentative pathway for alcohol production found in only *Cyanothece* 7424, 8801, and 8802.

**Conclusions:**

Our development workflow provides a path for constructing models using information from curated models of related organisms and reviewed gene annotations. This effort lays the foundation for the expedient construction of curated metabolic models for organisms that, while not being the target of comprehensive research, have a sequenced genome and are related to an organism with a curated metabolic model. Organism-specific models, such as the five presented in this paper, can be used to identify optimal genetic manipulations for targeted metabolite overproduction as well as to investigate the biology of diverse organisms.

## Background

Genome-scale models (GSMs) are the collection of gene to protein to reaction associations (GPRs), charge and elementally balanced reactions, and constraints on molecular functions found within a cell [1-4]. The constraints placed on molecular function define the possible phenotypes of an organism under specific conditions [2]. There are a number of applications for GSMs beyond the prediction of wildtype phenotypes in varying environments. These include the identification of optimal gene and medium modifications, non-native routes for metabolite production, and lethal gene deletions [5-9]. A genome-scale model of *Cyanothece* ATCC 51142, *i*Cyt773, was recently published [10]. It contains four compartments, with 811 metabolites and 946 charge and elementally balanced reactions. *i*Cyt773 is an improvement upon the previously published *i*Cce806 model [11], and contains 43 genes and 266 reactions unique from *i*Cce806 [10]. Further comparison of the two models can be found in the work by Saha et al. [10]. *i*Cyt773 also models the diurnal rhythm of the *Cyanothece* metabolism. Since *Cyanothece* ATCC 51142 is closely related to all five *Cyanothece* species discussed in this paper [12], it was used in the development of the reconstructions for five organisms, *Cyanothece* PCC 7424, 7425, 7822, 8801, and 8802, as *i*Cyc792, *i*Cyn731, *i*Cyj826, *i*Cyp752, and *i*Cyh755 respectively (all five models are included in Additional files 1 and 2). All models were named using their associated KEGG organism code. The objective of this study is to expediently generate models for a collection of members of a genus, using as a foundation an existing high-quality metabolic model for a representative member of the genus, while integrating information from a range of available sources.

The genus *Cyanothece* belongs to the phylum of Cyanobacteria. Cyanobacteria have a number of properties that make them ideal candidates for bio-production. Photosynthetic cyanobacteria bypass the need for sugar carbon substrates while having higher solar energy conversion efficiencies (i.e., 3-9%) than C3 (2.4%) and C4 plants (3.7%) [13]. *Cyanothece* generate not only hydrogen [12,14-16] but also fix atmospheric nitrogen by temporally segregating the photosynthesis and nitrogenase activities [17,18]. In addition, *Cyanothece* possess the potential to grow in air and can be easily fixed to solid matrices [19]. All five species discussed in this paper are capable of fixing nitrogen and producing hydrogen, while *Cyanothece* PCC 7425 is the only species that is not capable of accomplishing this task in an aerobic environment [12]. 7425 also varies in a number of physical characteristics, enough so that it has been suggested that it should be reclassified to another genus pending further review [20].

*Cyanothece* PCC 7424, 7425, 7822, 8801, and 8802, were all sequenced following the promising discoveries made concerning the metabolic capabilities of *Cyanothece* ATCC 51142 [12]. These five species exhibit unique metabolic characteristics that motivated the development of five separate reconstructions. Fragments of a butanol producing pathway have been postulated to exist in all strains through an inspection of the *Cyanothece* genomes [21]. Metabolic capabilities such as the alkane biosynthetic pathway and alternative pathways for breaking down arginine across species [12] have been hypothesized to exist as well. Given differences in metabolism, developed genetic systems [22], and variations in growth characteristics, phenotype, and rhythms of nitrogen fixation and respiration [23], it is important to globally assess the metabolic repertoire of each strain separately.

There exist numerous databases devoted to gene annotations for a wide variety of organisms [24-27]. However, the number of gene annotations is skewed towards a handful of extensively studied organisms. *Escherichia coli* K-12, the strain modeled in the *i*AF1260 metabolic reconstruction [28], has approximately 16 times the number of reviewed annotations (4,326) in the Universal Protein Resource (Uniprot) compared to *Cyanothece* PCC 7424 (271) [24]. For most (microbial) organisms Uniprot contains only a small subset of required gene annotations (i.e., 200–300). Faced with this paucity of organism-specific gene annotation information, most metabolic reconstructions rely on a single database (i.e., typically KEGG) from which to pull gene annotations [24,29-31]. This may introduce errors in the reconstruction as absent functionalities could be included in the model due to permissive homology cutoffs or errors in the original annotation source. In addition, specific and non-specific references to the same metabolite (e.g. D-Glucose vs. α-D-Glucose) and generic or unbalanced reactions [30] may also affect the consistency of the reconstruction. Integrating and contrasting information from multiple databases can remedy many of these shortcomings.

A systematic workflow is put forth that addresses the aforementioned challenges. It allows for the parallel reconstruction of genome-scale models for organisms that have a sequenced genome and are closely related to a species with a curated genome-scale model. Using this workflow, reconstructions were developed for all five *Cyanothece* species using *i*Cyt773 and reviewed annotations from Uniprot [24], NCBI Protein Clusters [32], and the Rapid Annotations using Subsystems Technology (RAST) method [33]. These annotations were used to retrieve charge and elementally balanced reactions from both the *i*Cyt773 model and the SEED database [34] for the construction of draft models. No reconciliation between the *i*Cyt773 and SEED reactions or metabolites was required as *i*Cyt773 was initially constructed using SEED notation when possible. The five models are all capable of producing biomass using the *i*Cyt773 biomass equations under diverse nutrient conditions. All five models are free of thermodynamically infeasible cycles, and the fractions of reactions mapped to specific genes (i.e., GPRs) are within the range of manually curated reconstructions. The use of multiple annotation sources helps to mitigate errors that may arise from a single source. Unlike automated draft models (i.e., Model SEED [35]), organism-specific metabolites such as pigments are included in the biomass equation and light reactions are fully traced. This workflow is also more adept at excluding metabolites present in related species but absent in the reconstructed organism. For example, menaquinone and ubiquinone are known to not exist within *Cyanothece*[36], but are often pulled into draft models generated by automated software.

## Results and discussion

### Model comparisons

**Figure 1:**
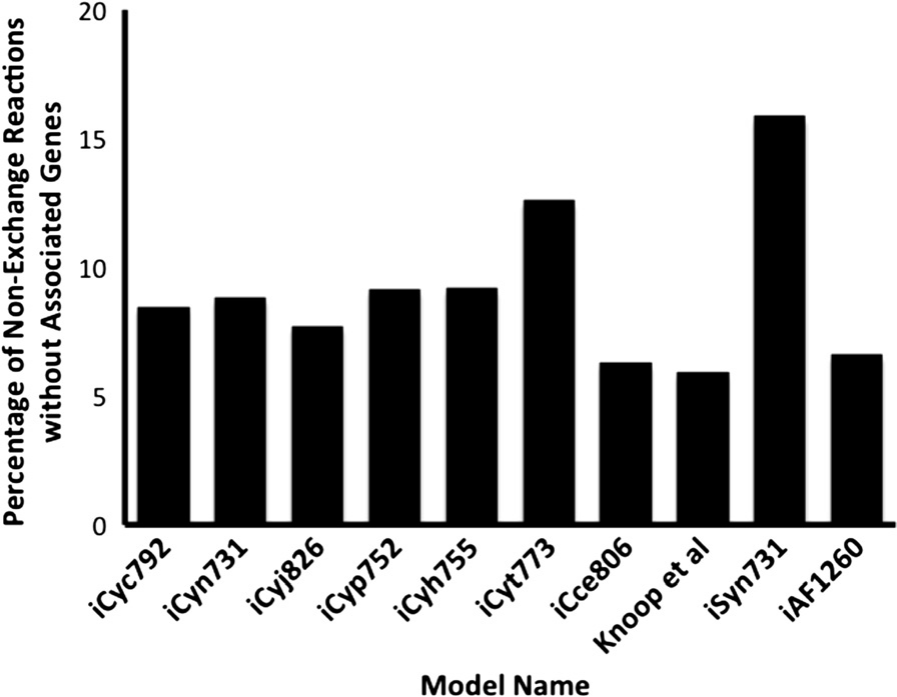
**Comparison of the percentage of non-exchange reactions without associated genes between the five models and five curated models, *i*Cyt773, *i*Syn731 **[10]**, *i*Cce806 **[11]**, *i*AF1260 **[28]**, and the *Synechocystis *PCC 6803 model developed by Knoop et al. **[37]**.** The model-organism correlations are *i*Cyt773 and *i*Cce806: *Cyanothece* ATCC 51142, *i*Syn731 and Knoop et al.: *Synechocystis* PCC 6803, and *i*AF1260: *Escherichia coli* K-12 MG1655.

The five models were developed by combining reactions from the curated metabolic model, *i*Cyt773, with reactions taken from the SEED database whose presence in that organism were confirmed by reviewed annotations. The statistics for the five developed models are shown in Table 1 (See Additional files 1 and 2 for model files). The model development workflow identified reactions that are in some cases unique to each reconstruction. However, closely related *Cyanothece* 8801 and 8802 have no unique reactions though they do contain a set of 30 reactions that are not found in any other reconstruction. All five models contain four compartments: cytosol, periplasm, thylakoid lumen, and carboxysome. The number of genes present in each reconstruction is similar to the number of open reading frames (ORFs) associated with the *i*Cyt773 and *i*Syn731 models. The percentage of non-exchange reactions without associated genes falls within ranges comparable to those of numerous manually curated models (see Figure 1) [10,11,28,37]. Biomass yields were also calculated for each of the five models using the same photoautotrophic conditions used to calculate the biomass yield for *i*Cyt773 [10]. All five models had an identical yield of 0.026 mole biomass/mole carbon fixed.

**Table 1:** Statistics for the five developed models: genes, reactions, and metabolites for each of the five models are listed, along with reactions that are unique to that reconstruction

	**Strain - reconstruction**				
	7424 -	7425 -	7822 -	8801 -	8802 -
	*i*Cyc792	*i*Cyn731	*i*Cyj826	*i*Cyp752	*i*Cyh755
Reactions	1242	1306	1258	1172	1161
Metabolites	1107	1160	1110	994	973
Genes	792	731	826	752	755
Unique reactions	41	149	40	0	0

**Figure 2:**
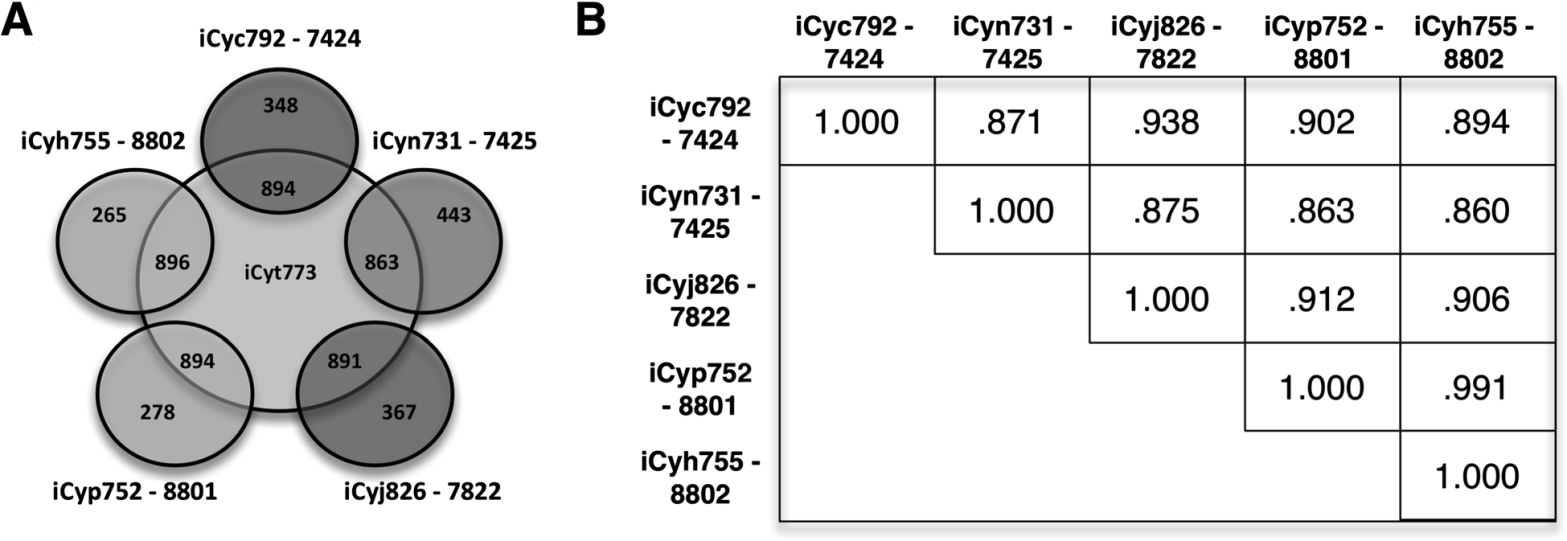
**Comparison of reaction similarity to phylogenetic relationships: (A) Venn diagram comparing the number of reactions each model shares with the *i*Cyt773 model (B) Similarity matrix for the five models.** See Methods for description of the similarity calculation done to compare reactions between two models. Both model names and organism numbers are included.

Figure 2 shows the number of reactions shared between *i*Cyt773 and each one of the models. A total of 922 reactions from *i*Cyt773 are shared with at least one of the five models while 169 reactions have been added to all five models during the SEED reaction retrieval step of the workflow. The removal of these 169 reactions only affects biomass production in *i*Cyn731. It does not grow when the reactions are removed since one of the reactions is essential as it is the only Fe(II) oxidoreductase present within iCyn731. The other four models contain another Fe(II) oxidoreductase. The number of reactions shared between each of the five models and *i*Cyt773 (Figure 2A) generally matches the phylogenetic relationships between the organisms [12]. *Cyanothece* 7425, which is the farthest removed of the five species from *Cyanothece* 51142, also has the fewest identified homologs with *Cyanothece* 51142. The two most closely related pairs, *Cyanothece* 7424/7822 and 8801/8802, have the highest reaction similarities (see Figure 2B) while the farthest removed species, *Cyanothece* 7425, has the lowest similarity. This divergence lends support to the possibility of reclassification [20].

### Model validation using published findings

The effect of a gene knockout on an organism’s phenotype is frequently used in assessing GSM quality [10,38]. However, unlike the CyanoMutants database for *Synechocystis* PCC 6803 [39,40], none of the five species have a detailed repository of known mutants. The Δ*nifK* mutant for *Cyanothece* 7822 was shown to not be able to grow without the presence of combined nitrogen (nitrate) [22]. This finding implies the critical involvement of *nifK* in the fixation of nitrogen. In *i*Cyj826 this gene is involved in the GPR of the nitrogen fixation reaction present within the model. Given that the GPR describes *nifK* as one of three critical subunits of the enzyme, its deletion results in the inability for that reaction to carry flux. Upon its removal from *i*Cyj826, the model is unable to grow without the addition of nitrate or ammonium, showing that the model reacts to the knockout in the same manner as the organism does *in vivo*.

Despite the many similarities between the five species, significant differences also exist [12]. Genes that code for isocitrate lyase and malate synthase (glyoxylate shunt) are present only in *Cyanothece* 7424 and 7822 as reflected in the models. 2-oxoglutarate decarboxylase and succinic semialdehyde dehydrogenase, found in many cyanobacteria, complete the TCA cycle despite the absence of 2-oxoglutarate dehydrogenase [41]. Both of the enzymes in the alternate pathway are present within *i*Cyt773, and were transferred to all five models. The associated genes are also bidirectional best hits with the two genes in *Synechococcus* PCC 7002 that are associated with the aforementioned enzymes [41]. *i*Cyn731, *i*Cyp752, and *i*Cyh755 all contain an alkane biosynthetic pathway similar to what is present within *i*Cyt773. While *i*Cyt773 contains the pathway that enables the production of pentadecane from hexadecenoyl-ACP, Schirmer et al. have measured heptadecane but not pentadecane production from *Cyanothece* 7425 [42]. *i*Cyn731 contains only heptadecane production, while *i*Cyp752 and *i*Cyh755 contain pathways for both pentadecane and heptadecane (no specific literature evidence neither in support nor in conflict with this was found). The two enzymes required, hexadecenoyl-ACP reductase and hexadecenal decarbonylase (enzyme commision (EC) numbers 1.2.1.80 and 4.1.99.5 respectively per *i*Cyt773), have no corresponding annotations or orthologous genes in *Cyanothece* 7424 or 7822 [42].

Polyhydroxyalkanoates (PHAs) are a complex family of polyesters that can be synthesized by a wide variety of bacteria [43]. *Cyanothece* 7424, 7425, and 7822 all contain the enzymes keto-thiolase and acetoacetyl-CoA reductase, which are necessary for the synthesis of polyhydroxyalkanoic acids [43-45]. There are RAST and unreviewed Uniprot annotations that identify genes within each of these three organisms associated with a PHA synthase. The non-fermentative pathway for higher alcohols exist in the 7424, 8801, and 8802 strains [12]. The same pathway has been seen in *E. coli*[46,47] after the addition of the *kivD* gene from *Lactococcus lactis*[48] and the *adh2* gene from *Saccharomyces cerevisiae*[49]. The pathway uses the 2-keto acid intermediates of amino acid biosynthesis and diverts them towards the synthesis of alcohols [46]. The *kivD* gene codes for a 2-keto acid decarboxylase that acts on a wide range of substrates and enables the conversion of the 2-keto acids into aldehydes. The workflow identified genes in *Cyanothece* 7424, 8801, and 8802 which are bidirectional best hits with the *kivD* gene from *Lactococcus lactis*, and also annotated as being associated with the same EC number as *kivD*. An alcohol dehydrogenase, such as *adh2*, then converts these aldehydes into alcohols. The *adhA* gene (slr1192) in *Synechocystis* PCC 6803 has been found to have wide substrate specificity that includes the aldehydes associated with butanol and propanol [50]. All five species contained a gene that was a bidirectional best hit with slr1192. While both the forward and reverse BLAST searches for *Cyanothece* 7425 had e-values on the order of 10^-28^ and percent identities of 30%, the searches, both forward and reverse, for the other four organisms had e-values ranging between 10^-138^ and 10^-153^ with percent identities ranging from 58 to 61%. The presence of orthologs to both a 2-keto acid decarboxylase and alcohol dehydrogenase with wide ranges of specificity in *Cyanothece* 7424, 8801, and 8802 provides annotation evidence for the hypothesized presence of non-fermentative higher alcohol pathways [12].

Significant variations in nitrogen metabolism between the five species has been documented [12]. Arginine decarboxylase is present in all five reconstructions, but differences arise in the subsequent agmatine catabolism. *Cyanothece* 51142 does not contain the associated genes for the conversion of agmatine to putrescine, and this is reflected in the *i*Cyt773 model [10,12] as these reactions are absent. Both *i*Cyc792 and *i*Cyj826 contain agmatinase and urease. The proposed pathway for agmatine breakdown into putrescine in *Cyanothece* 7425, 8801, and 8802 is through N-carbamoylputrescine. The two reactions required for this degradation can be found in all three associated models. Finally, as predicted by Bandyopadhyay et al. [12], *i*Cyc792, *i*Cyj826, *i*Cyp752, and *i*Cyh755 contain the reactions required to break putrescine down into spermidine and spermine.

### Validation of proposed reconstruction workflow

**Figure 3:**
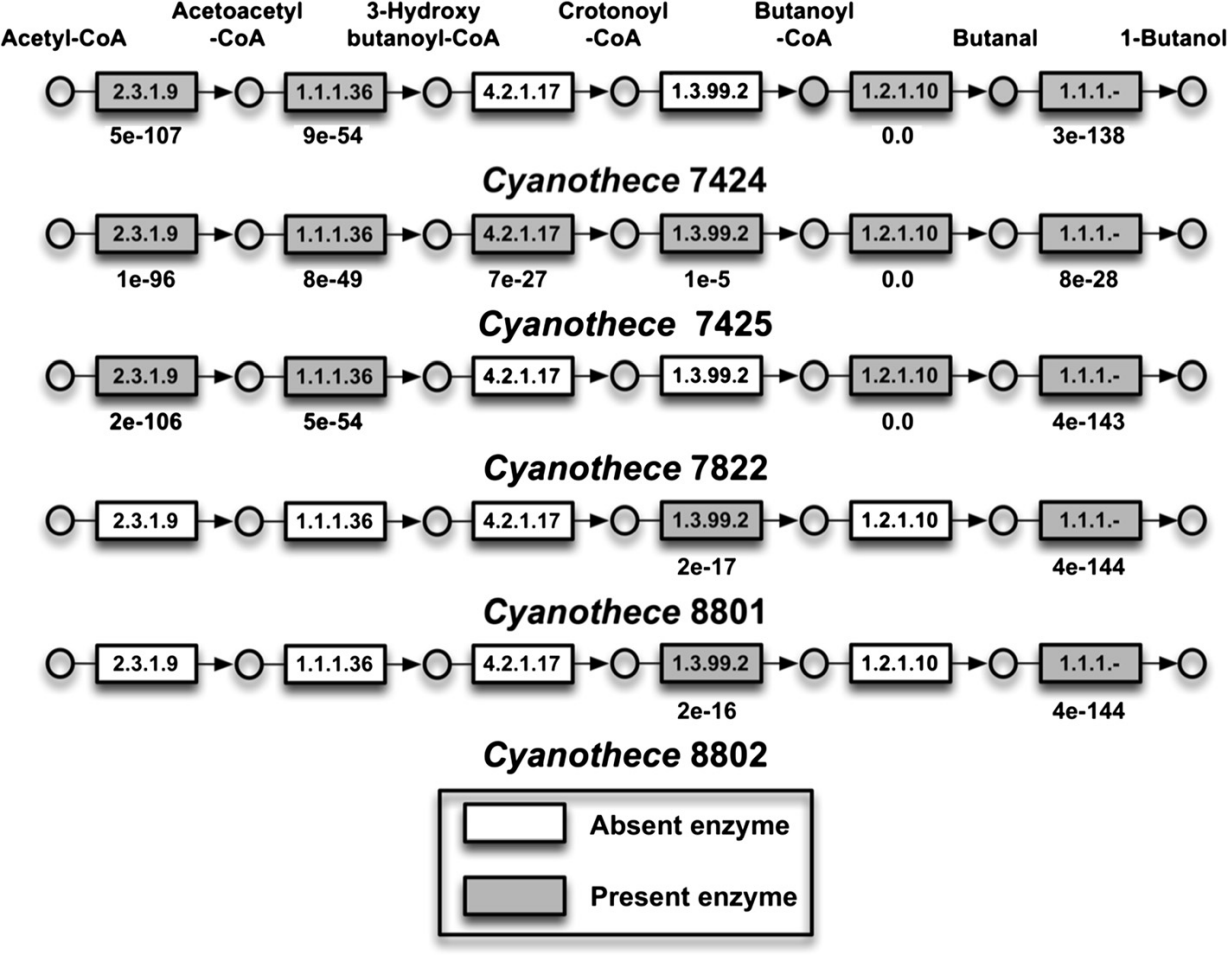
**Comparison of fermentative butanol pathway enzymes present in each of the five species: The enzymes highlighted are present in the organism’s reconstruction along with the associated reaction.** Listed e-values are for BLAST searches between the genes of the five species and the associated gene in *Clostridium acetobutylicum* and the *adhA* gene in *Synechocystis* 6803. EC-gene relationships: 2.3.1.9: CA_C2873, 1.1.1.36: CA_C2708, 4.2.1.17: CA_C2712, 1.3.99.2: CA_C2711, 1.2.1.10: CA_P0035, 1.1.1.-: slr1192.

Additional reactions retrieved using reviewed annotations have provided a number of insights into the five species that would not have been either found or confirmed if reactions were only pulled from *i*Cyt773. The diverging nitrogen metabolism reactions were retrieved using SEED, as agmatine is the preferred polyamine for *Cyanothece* 51142 [12]. An alternative butanol pathway is present in varying stages of completion in the five models. While butanol can be produced from a 2-keto acid as previously discussed, it can also be produced through the coenzyme A dependent pathway [51,52]. The coenzyme A dependent pathway was found to exist within a *Clostridium* species [53,54]. Figure 3 shows the comparative level of completion of the fermentative butanol pathway within each of the five species. *Cyanothece* 7425 is the only organism to contain the complete pathway. The alcohol dehydrogenase exists within the models given the identification of homologs to the *Synechocystis adhA* gene [50]. The 7424/7822 and 8801/8802 pairs have the same enzymes. Figure 3 also shows e-values for the BLAST searches between the genes and the genes in the fermentative butanol pathway of *Clostridium acetobutylicum* ATCC 824. Given the lower e-value for Butanoyl-CoA dehydrogenase, it is the gene most likely to not be present or functional within *Cyanothece* 7425. The enzymes present in the five pathways mirror the phylogenetic relationship trends of the five species in a manner comparable to what was initially seen in the reaction similarities from Figure 2, as well as with the glyoxylate shunt and nitrogen metabolism.

The proposed workflow also served to complete unfinished pathways from *i*Cyt773. All five models are capable of converting galactose-1-phosphate to fructose-6-phosphate as in *i*Cyt773. Three of the models, *i*Cyn731, *i*Cyj826, and *i*Cyh755, also include the reaction that converts galactose into galactose-1-phosphate, enabling them to process galactose in the glycolysis pathway. Tetrahydrobiopterin (BH4) is a pteridine compound that acts as a cofactor for nitric oxide synthases and aromatic amino acid hydrolases in higher animals [55]. Pteridine glycosides have been found in cyanobacteria, although their function is still unknown [56], and the first isolated pteridine glycosyltransferase from *Synechococcus* PCC 7942 acted on BH4 [57]. Even though *i*Cyt773 does not contain the complete BH4 pathway as described by Thony et al. [55], our workflow completed the pathway in all five species, identifying a gene that is a bidirectional best hit with the gene in *Synechococcus* PCC 7942. The reaction was not included in the models, as it does not exist within the SEED reaction database. All enzymes that were retrieved from annotations but were not included in the model because of a lack of associated reaction in the subset of the SEED database used for model development are listed in Additional file 3.

Reactions not transferred from *i*Cyt773 offer insight into divergences between the metabolism of the new organism and the reference model. Two of the reactions that were not transferred from *i*Cyt773 to the models for *Cyanothece* 7424 and 7822 are responsible for the conversion of hexa- or octadecenoyl-ACP to n-hepta or pentadecane. As previously mentioned it is accepted that the alkane biosynthetic pathway does not exist in these organisms [42]. Another compound that is generally not found in the five species is xanthine, a purine base involved in the breakdown of purine ribonucleotides such as inosine-5′-phosphate and xanthosine-5′-phosphate, into uric acid. *i*Cyt773 can produce xanthine from either hypoxanthine or xanthosine, *i*Cyc792 only contains the reactions for production from xanthosine and cannot break xanthine down into uric acid. *i*Cyn731 only contains the reactions for production from hypoxanthine, but can convert xanthine into uric acid. The other three species do not contain any reactions involving xanthine and thus cannot process purine ribonucleotides through this pathway. Six reactions involved in transporting metabolites between the cytoplasm and periplasm or extracellular space were not transferred, such as molybdate transport via the ABC system. Given the likelihood that such reactions still exist within the other *Cyanothece* strains, it is possible that the associated GPR in *i*Cyt773 should be reevaluated for these reactions.

### Comparisons with other model development methods

Current model development methods can be generally characterized as manual, semi-automated, or automated. The workflow presented in this paper is best classified as semi-automated. This workflow allows for more expedited model development while avoiding some of the sources of error plaguing automated model generation and allowing for a wide range of customization. This workflow can be adapted for use with any models, annotation sources, and additional reaction sets given annotation availability and user preferences.

Many draft models are nowadays generated through the identification and comparison of homologs with the GPRs of curated models [58-61]. Hamilton et al. identified the possibility for bidirectional BLAST searches to identify false positive ortholog pairs [61]. The e-value cutoff for the searches performed for the test was 10^-5^. Here we use a more conservative cutoff of 10^-30^ to safeguard against such instances. When the cutoff was relaxed from 10^-30^ to 10^-5^ for the bidirectional BLAST between *Cyanothece* 51142 and the five species there were between 280 and 403 additional best hit pairs for each of the organisms. The number of these pairs that involved genes present in *i*Cyt773 varied between 15 for *Cyanothece* 7424 and 8801, and 26 for *Cyanothece* 7425. The reliance of manually constructed models on reviewing every annotation and manually curating the model greatly increases the time spent on development. This workflow helps to mitigate the need for manual review of each annotation by only using annotations that are reviewed or are derived from reviewed sources. Manual curation can then be reserved for certain key steps. Some of these models only include additional reactions beyond those retrieved from the curated models if the reactions are required for biomass production [58,60,61]. This restricts the inclusion of reactions unique to either that organism or a subset of organisms that the reference models do not belong to. This introduces the risk of not including secondary metabolism pathways, which could be of great interest. The workflow presented here aims to overcome this through the use of outside annotations to retrieve SEED reactions.

There are a number of approaches for the automated development of metabolic reconstructions [35,62-64] affording significant gains in development time, however, at the expense of some omissions and erroneous additions. The *Cyanothece* models created using the MIRAGE method contain generalized lipids along with a non-specific acceptor metabolite [64]. Both the KBase and MIRAGE models constructed for *Cyanothece* 7424 contain menaquinone and ubiquinone, compounds shown to not exist within that organism [36]. Conversely, there are a number of metabolites present in the biomass composition of the five reconstructed models that do not exist within either in the KBase or MIRAGE models (i.e., 22 specific lipid metabolites, 4 pigments and cyanophycin). The model produced through KBase also does not contain the pigment β-carotene. Many of these models do not have specified compartments apart from cytoplasm and extracellular space [35,62,64]. Automated model development can exclude unique metabolic pathways if they are absent from the training set of models. Specifically, both the MIRAGE and KBase models generally lack light reactions.

Other methods that combine manual and automated steps provide their own distinct approach to model reconstruction. The RAVEN toolbox [65] allows for the curation of a reconstruction from models of related species using homologs identified through BLAST bidirectional best hits, and additional unique functions supplied through annotations taken from KEGG Orthology [26]. This method was employed for the construction of the *Penicillium chrysogenum* model *i*AL1006 [65]. Our workflow can currently pull from up to three sources, with the ability to quickly expand the number of sources sampled, resulting in more identified EC numbers with higher confidence.

## Conclusions

In this paper we presented a workflow that was used to rapidly develop curated models for five *Cyanothece* strains using the previously published *i*Cyt773 model and reviewed annotations from numerous sources. The comparisons between these five models line up with the established phylogenetic relationships between the species. Specific reactions that were both kept from being taken from *i*Cyt773 or added from the SEED database demonstrate the efficacy of this workflow and provide insights into the metabolism of the five species, as well as suggesting areas for further curation in the *i*Cyt773 model. This workflow can easily be adapted to work with other metabolic models, annotation sources, and reaction databases. All five models (*i*Cyc792, *i*Cyn731, *i*Cyj826, *i*Cyp752, and *i*Cyh755) are included in the supplementary material.

## Methods

### Draft model development

**Figure 4:**
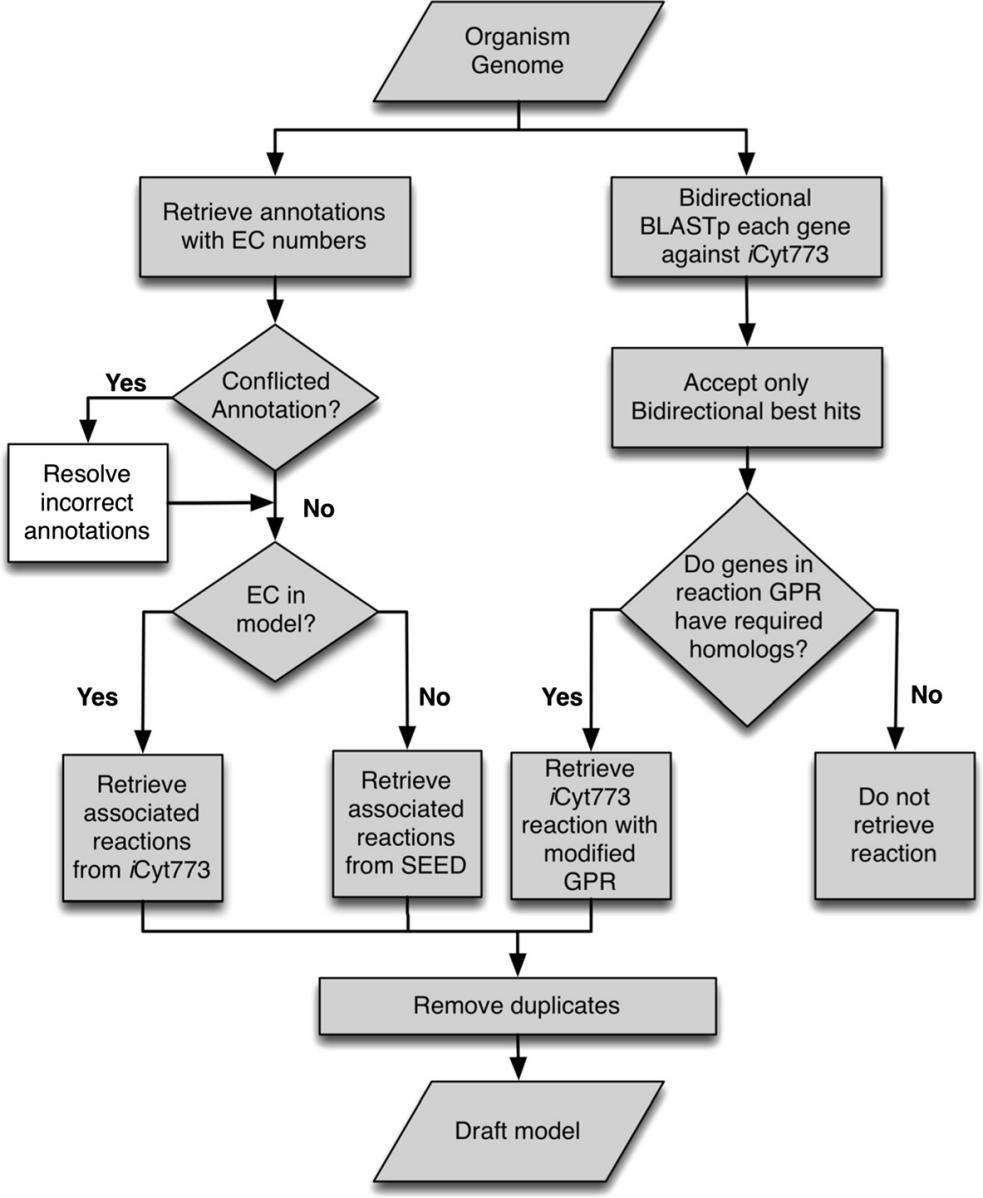
**Workflow for development of draft models: These models are developed from a sequenced genome and curated genome scale model of related organism.** The right hand side outlines the steps required to evaluate the reactions in *i*Cyt773 for their presence in the other organisms. The steps to retrieve gene annotations and resolve any conflicts are shown on the left hand side. The steps in gray were automated, whereas the manually performed step, the resolution of conflicting annotations, is shown in white.

Draft models for the five organisms were developed using the workflow shown in Figure 4, which uses a combination of reviewed gene annotations and identified homologs between the new organism and *Cyanothece* 51142. Reactions that were determined to exist in both *Cyanothece* 51142 and the organism being modeled were transferred from *i*Cyt773 to the draft model. This reaction sharing was established through a comparison of homologs between the two genomes. These homologs were determined using a bidirectional BLAST search between the genomes of *Cyanothece* 51142 and the organism, using an e-value cut off of 10^-30^ and the requirement of mutual best hits. The Boolean logic given by each GPR in *i*Cyt773 was evaluated using these bidirectional hits. If the organism contained the homologs required to satisfy the logic and encode the protein, the reaction was transferred to the draft model. This only requires one isozyme to be present within the organism (i.e. if the associated genes for a reaction are listed as “gene A OR gene B OR gene C”, only one of the three genes must have a homolog), yet requires that all genes that code for an essential protein complex have a homolog. These identified reactions were added to the draft model with the GPRs modified to reflect the homologs present in the organism. Both the homology searches and identification of reactions to be included in the model were automated steps.

Reviewed annotations retrieved from Uniprot [24], NCBI Protein Clusters [32], and RAST [33], are used to support the inclusion of additional reactions into the draft models. An automated process was used to retrieve annotations that reference specific EC numbers, along with the EC numbers associated with the reactions retrieved using bidirectional BLAST. Only specific EC numbers were used to avoid the inclusion of unnecessary reactions. For some genes the annotations are inconsistent. These discrepancies are resolved through a manual multi-step procedure shown in Figure 4. First the EC numbers are checked to confirm that they have not been transferred to a new number. An example of this transfer of EC numbers can be seen with the annotations for the *Cyanothece* 7424 gene PCC7424_1895. Both Uniprot and NCBI Clusters assigned the EC 2.5.1.75 to the gene, whereas the RAST method assigned the EC number 2.5.1.8. Despite the apparent mismatch, EC 2.5.1.8 had previously been transferred to 2.5.1.75, resolving any conflict between the annotations. If the enzymes are uniquely classified, a search of literature, specifically the InterPro database [66], is then performed to validate their existence (or non-existence) in the organism. The *Cyanothece* 7424 gene PCC7424_2477 has an associated annotation of 1.1.1.29 from *i*Cyt773, whereas RAST assigns both 1.1.1.26 and 1.1.1.81 to the gene. InterPro states that the 1.1.1.26 enzyme belongs to a protein family that is found in hyperthermophilic archaea, thus ruling out its existence in *Cyanothece* 7424. After using the InterPro information to rule out a possible associated enzyme, the annotation is resolved through order of confidence (described below), and 1.1.1.29 is attributed to the gene. Next, any enzymes that are associated with generic metabolites, or metabolites known to not be found within the organism, are removed. Such filtering can be seen with the *Cyanothece* 7425 gene Cyan7425_1569. Both the model and RAST annotation suggest that succinate dehydrogenase (1.3.99.1) is associated with this gene. However NCBI Protein Clusters suggests enzyme 1.3.5.1, which is a succinate dehydrogenase specific to ubiquinone. As ubiquinone is not present within *Cyanothece*[36], this conflict is resolved. The list of all reactions removed from each model for containing generic metabolites is included in Additional file 4. If discrepancies still exist, annotation resolutions are made based on a confidence order of *i*Cyt773, Uniprot, NCBI, and RAST. The order of confidence is derived from the likelihood that a source has been manually reviewed and is applicable to the individual gene in question. *i*Cyt773 GPR relationships were curated specifically for a *Cyanothece* model. Uniprot reviewed annotations are manually annotated individually [24], while the protein cluster annotations used in this study are curated as a group of related genes [32], and RAST annotations are developed using the manually curated FIGfams [33,67]. Lower confidence is placed in these annotations, as it is possible that the automated RAST program could improperly assign annotations in some cases. If all of the enzymes proposed by the other annotation sources are contained within the list of enzymes found to relate to the gene through inspection of *i*Cyt773, the annotation is not listed as conflicting and the enzymes from the model are used. There were on average between 40 and 50 genes with conflicting annotations. Between 55 and 70% of conflicts required order of confidence to resolve. Using multiple sources allows for the identification of probable errors in the databases. These annotations can also reveal errors in other databases not used in the model development. One such example is gene PCC7424_2817 in the *Cyanothece* 7424 genome. All sources used in this paper, along with KEGG Orthology [27], indicate that the enzyme associated with this gene is 2-succinyl-5-enolpyruvyl-6-hydroxy-3-cyclohexene-1-carboxylic-acid synthase (EC 2.2.1.9). Both the KEGG and REFSEQ [68] annotations list the same enzyme name, but list the EC number as 4.1.1.71 (associated with 2-oxoglutarate decarboxylase).

Subsequently, this resolved list of EC numbers is referenced against the *i*Cyt773 model. Reactions with a matching EC number are retained, and the remaining EC numbers are used to retrieve reactions from the SEED database [34]. Reactions are only taken from the subset used by the SEED service for GapFilling [69], as these reactions are confirmed to be charge and elementally balanced. Those EC numbers that did not have an associated reaction within this set of SEED reactions and were therefore not included within the models are compiled in Additional file 3. All duplicate reactions retrieved from *i*Cyt773 are removed while the remaining reactions necessary for photosynthesis are included. These reactions are known to exist within the organisms, as they can grow autotrophically. Any oxidative phosphorylation reactions or diffusion transport reactions that had not previously been added to the model are appended given their obvious essentiality. This set of reactions constitutes the draft model. All steps in draft model development are automated except for the EC annotation reconciliation. The time required to complete this step is reduced as more models are developed, and results can be applied to related organisms.

### Biomass and removal of thermodynamically infeasible cycles

The four biomass descriptions developed for the *i*Cyt773 model were used in the five models [10]. Initially, all draft models were not capable of producing biomass. A subset of reactions from iCyt773 needed to be included in the draft models to allow for the generation of biomass. A mixed integer linear program was used to determine the minimal set of additional reactions required for the production of biomass. All alternative solutions within two reactions of the global minimum were found, and every reaction was examined for evidence suggesting its existence within the organism. Given the necessity of their inclusion for biomass production even reactions with no identified evidence were included in the models. In situations with several alternate solutions, the solution that contained the most reactions with evidence for their inclusion was chosen. Necessary reactions, which could not have previously been included in the models as they did not have associated enzymes or genes, were added at this point. Between three and eight reactions with a GPR in *i*Cyt773 that did not have direct literature or annotation evidence were included in order to produce biomass. A substantial number of these reactions did not have both a gene and enzyme associated in *i*Cyt773, which would lower their chance to be included during the initial stages of draft model development (See Additional file 5 for a full list of reactions included in this step). While the initial reaction set was generated for the production of 1% of the maximum biomass when all *i*Cyt773 reactions were included, the inclusion of two reactions expected to be present in all models, the exchange reaction for oxygen and the diffusive transport of carbon dioxide between the periplasm and cytoplasm, allowed for biomass production exceeding 90% of the maximum. The 7425 model requires an additional two reactions to produce maximum biomass, but the other four models are capable of such production with the addition of the carbon dioxide transport and oxygen exchange reactions. This process was performed for both autotrophic and heterotrophic growth conditions. For autotrophic growth, 16 reactions were added to *i*Cyc792*,* 24 to *i*Cyn731, and 18 to *i*Cyj826, *i*Cyp752, and *i*Cyh755. The same approach was used for heterotrophic growth, where only *i*Cyn731 required the inclusion of one reaction to grow under heterotrophic conditions.

The models were further modified to avoid the presence of thermodynamically infeasible cycles. Flux variability analysis was performed to identify unbounded reaction fluxes. Given the absence of thermodynamically infeasible cycles within *i*Cyt773, added reactions from SEED were solely responsible for the creation of any cycles. The number of SEED reactions present in cycles varied between 39 in *i*Cyh755 and 51 in both *i*Cyn731 and *i*Cyj826. Three steps were taken to modify the SEED reactions involved in the cycles. First the Gibbs free energy values provided by SEED were examined. Any reactions where the entire free energy value range, factoring in error, was more than 4 kcal/mol removed from zero was restricted to the directionality specified by Gibbs free energy. Any SEED reactions whose fluxes still hit the bounds were restricted to the direction opposite of the cycle. The annotations of the few SEED reactions that were still involved in cycles were inspected. All of these reactions were supported solely by RAST annotations. Given this lower confidence due to the single-source annotation, the reactions (between four and ten for each model) were removed. Additional file 6 lists all reaction modifications made to eliminate the cycles.

### GPR development

GPR relationships were primarily derived from either the previous bidirectional BLAST analysis of *i*Cyt773 reactions or the analysis of retrieved annotations. Bidirectional best hits were previously used to evaluate the presence of each reaction in the new organism. If a reaction is added to the model, the GPR for every isozyme or complete subunit that is present is translated to the list of genes for the new organism.

The GPR relationships for reactions retrieved from SEED were developed by applying the Autograph method [70]. All genes that were linked to an enzyme through an annotation were used for the GPR for each reaction associated with that enzyme. If there are RAST annotations for each of these genes with the correct EC annotation, then they are used for the comparison. For all five species there were no ECs for which this was not the case. Genes that shared the same annotation designation were determined to be isozymes while those with different names were seen to be subunits of a protein. There is a small subset of reactions in the models that were taken from *i*Cyt773 because of either proof of their existence (e.g. photosynthetic reactions) or their requirement for biomass production. Many of these GPR relationships are missing a small number of bidirectional best hits. For these genes the BLAST cutoff was reduced to 10^-10^. These few additional best hits aided in the resolution of many of the remaining reactions, leaving between six and thirteen of the reactions without a transferred GPR.

### Model simulations and analysis

Flux balance analysis [71] was used in both the model development and model validation phases to determine flux distribution under varying conditions.

*Maximize v*_*Biomass*_

Subject to

(1) $$\sum_{j=1}^{M}S_{\mathrm{ij}}v_{j}=0,\forall i\in 1,\ldots ,N$$

(2) $$v_{j,\text{min}}\leq v_{j}\leq v_{j,\text{max}},\forall j\in 1,\ldots ,M$$

Where S_ij_ is the stoichiometric coefficient for metabolite i in reaction j, v_j,min_ and v_j,max_ denote the minimum and maximum flux values for reaction j, while v_j_ represents the flux value of reaction j. N and M denote the total number of metabolites and reactions respectively.

A mixed integer linear program was used in the determination of a minimal set of reactions necessary for biomass production using the following formulation.

$$\mathrm{Minimize}\;\sum_{j}^{M}y_{j}\;$$

Subject to

(3) $$\sum_{j=1}^{M}S_{\mathrm{ij}}v_{j}=0,\forall i\in 1,\ldots ,N$$

(4) $$v_{j,\min}y_{j}\leq v_{j}\leq v_{j,\max}y_{j},\forall j\in 1,\ldots ,M$$

(5) $$v_{\mathrm{Biomass}}\geq v_{\mathrm{Biomass}}^{\min}$$

All reactions were assigned a binary variable y_j_, which when equal to zero eliminates flux through reaction j. The value of y for all reactions present in the draft model was fixed at one. Biomass production was fixed at greater than 1% of the maximum value when all *i*Cyt773 reactions were included, and the number of included reactions was minimized.

Flux variability analysis was used for identification of reactions present within cycles, and used the following formulation.

(6) $$\left[\begin{array}{c} \text{Max}/\text{Min}\;v_{j}\\ \text{Subject}\;\mathrm{to}\\ \sum_{j=1}^{M}S_{\mathrm{ij}}v_{j}=0,\forall i\in 1,\ldots ,N\\ v_{j,\min}\leq v_{j}\leq v_{j,\max},\forall j\in 1,\ldots ,M \end{array},\;\right]$$

(7) ∀j∈1,…,M

No constraints were placed on the biomass growth so as to identify all possible cycles within the model. This analysis was performed iteratively after each series of modifications was made to the reactions present within the cycles.

The reaction similarity between any two models is calculated using the following formula,

(8) $$\text{Similarity}=\sqrt{\frac{A^{2}}{\left(A+B\right)\left(A+C\right)}}$$

A denotes the total number of shared reactions between the two organisms, whereas B and C represent the number of unique reactions in each model.

CPLEX solver (version 12.3 IBM ILOG) was used in the GAMS (version 23.3.3, GAMS Development Corporation) environment for solving the optimization models. All computations were carried out on Intel Xeon X5675 Six-Core 3.06 GHz processors that are a part of the lionxf cluster, which was built and operated by the Research Computing and Cyberinfrastructure Group of The Pennsylvania State University. All codes used in model development were written using the Python programming language.

## Competing interests

The authors declare that they have no competing interests.

## Authors’ contributions

TJM designed model development workflow and developed models; TJM, BMB, HBP, and CDM analyzed the data; TJM and CDM wrote the paper. All authors read and approved the final manuscript.

## Supplementary Material

Additional file 1 **All five genome-scale reconstructions (*i*Cyc792, *i*Cyn731, *i*Cyj826, *i*Cyp752, and *i*Cyh755), along with GPR and metabolite information.** Click here for file

Additional file 2 **A zipped file containing all five genome-scale reconstructions (*i*Cyc792, *i*Cyn731, *i*Cyj826, *i*Cyp752, and *i*Cyh755), along with GPR and metabolite information in SBML format.** Click here for file

Additional file 3 List of retrieved EC numbers which were not associated with any reactions in the SEED subset of reactions used for draft model development. Click here for file

Additional file 4 List of reactions removed from the five models for containing generic metabolites. Click here for file

Additional file 5 Set of reactions added to each model to insure biomass production, along with associated support for inclusion. Click here for file

Additional file 6 All reaction modifications made to eliminate thermodynamically infeasible cycles. Click here for file
